# Eye tracking and child sexual offenders: a systematic review

**DOI:** 10.1080/20961790.2021.1940737

**Published:** 2021-07-30

**Authors:** Tony Godet, Gérard Niveau

**Affiliations:** Unit of Forensic Psychiatry, University Center of Legal Medicine, Geneva University Hospitals, Geneva, Switzerland

**Keywords:** Forensic sciences, forensic psychiatry, eye tracking, paedophiles, sexual offenders, systematic review

## Abstract

Eye tracking is used in sexology to identify attractiveness and sexual desire indirectly. This systematic review summarizes results of works that have used eye tracking to analyse paedophilic interest in order to investigate its potential as a useful forensic tool. Six studies met the inclusion criteria. Five of them concerned a large study project and used approximatively the same sample of paedophiles (inpatients), forensic patients (without a sexual interest in children) and healthy controls to make comparisons between the three groups. One study added 11 self-declared paedophiles (outpatients) for a comparison between inpatient paedophiles, outpatient paedophiles and controls (healthy and forensic inpatients). One study compared a group of child sexual offenders with non-offenders. All studies used static pictures of male and female subjects at different pubertal stages. Some studies divided every picture into a different area of interest. Dependent variables used are fixation latency (early attention) and relative fixation time, (later attention). Each study identified significant differences between the paedophile group *versus* other groups: shorter fixation latency on child pictures, longer fixation time on child pictures and number of fixation most important on child pictures. Two scores (age preference index and attentional control index) showed hight and/or moderate sensitivity and specificity. Although the results suggest the eye tracking can discriminate between paedophile interest and non-paedophile interest, there are too few studies on this specific topic and further research is needed with larger and different sample, carried out by different research teams. If these findings were confirmed, it remains unclear as to their impact in a forensic context when presented openly in Court.

Key points

- Eye tracking identify attractiveness and sexual desire indirectly.
- Eye tracking may discriminate between paedophile and non-paedophile subjects.
- The use of eye tracking in forensic contexts should respect some ethical concerns to avoid drifts.

## Introduction

Sexual assaults are a substantial social and public mental health concern. Official statistics have reported approximately 5 000 sexual assaults (with or without contact) in 2012 in Quebec (Canada), approximately 580 000 declared by victims in France in 2016, and more than 8 000 offences against sexual integrity reported in 2019 in Switzerland [1–3]. An important number of these victims are children. Health consequences associated with sexual abuse in children are very important, physical and psychological, as low self-esteem, symptoms associated with post-traumatic stress disorder, substance abuse, etc. [4–6]. Legal pursuits are often initiated. These types of acts ask about a possible diagnosis of paedophilia of alleged offenders [7,8].

Paedophilia is a psychiatric disorder of sexual preference, it is a paraphilia. Its definition slightly evolved through the new international classifications. The International Statistical Classification of Diseases and Related Health Problems 10 (ICD-10) defines this disorder as: “A sexual preference for children, boys or girls or both, usually of prepubertal or early pubertal age” [9]. To have paedophilic interests is insufficient to diagnose a paedophilic disorder. Similarly, the Diagnostic and Statistical Manual of Mental Disorders 5 (DSM-5) defines a paedophilic disorder as a recurrent sexual interest in prepubescent children (generally aged 13 or younger) ­characterized by persistent thoughts, fantasies, urges, sexual arousal or behaviour [9,10]. One of the most important risk factors of recidivism of child abuse is the presence of a paedophilic disorder and there is a strong correlation between child sexual assault and a sexual preference for children [8,11–13]. In fact, 50% of convicted child sexual offenders had sexual preference for children [8,14,15]. However, paedophilia is difficult to diagnose with accuracy, particularly in the forensic context, and especially when the diagnosis is based on self-report data [9,10,16,17]. In the psychiatric assessment context, subjects rarely self-report their sexual preference for children, particularly if proof or evidence are unclear.

To improve the accuracy of this diagnosis, an objective measure of male sexual arousal was ­developed in the 1950s, named “penile plethysmography” (PPG) [18]. Since then, several studies have been conducted—some of which are relatively recent—and some researchers view the PPG as the gold standard of male sexual arousal measurement [19–22]. Many psychiatric forensic departments or clinics use PPG in forensic assessment, especially in North America [23,24]. In Europe, this measurement has been criticized and its reliability questioned, especially in the forensic context with “no voluntary” subjects compared with subjects included in studies [25–27]. Other researchers have raised the possibility that some subjects may fake responses with PPG through a voluntary control of their erectile response [28]. Due to this, it is estimated that the sensitivity is approximately 60%, which is moderate [29–31]. Moreover, the lack of standardization of procedures and materials has also been questioned [28,32]. In order to reduce fake responses and increase the sensitivity of PPG, researchers add tasks, such as semantic identification or eye tracking, to control volunteer erectile inhibition during PPG procedure [24,33].

Eye tracking has been used in the psychological field since late 19th century, but only recently with regard to sexology [34–36]. This technique is directly linked to ocular movement and allows a direct observation of early attention (initial orientation) and late attention (maintenance of attention) and the detection of various stimuli, in real time [37]. Early attentional processing was assessed by the first fixation and the first fixation duration after presentation of the stimulus, and late attentional processing was assessed by relative fixation time. Several studies showed, at least to some extent, that attentional processes are automatic and cannot be controlled consciously [38]. Eye movements are recorded with a camera, often by infrared light, thus allowing to determine the direction of the gaze. In sexology, the first research study focused on gender differences using erotic and non-erotic stimuli [36, 39,40]. Eye movements differ between erotic and non-erotic stimuli, men and women visually attended to the body more in the erotic stimuli than in the non-erotic stimuli (greater number of fixations and longer total time devoted to that region) [39,41,42]. Moreover, sexual orientation had an influence: ­heterosexual men looked significantly more often at female than at male pictures, whereas heterosexual women looked equally at male and female pictures [41]. One of the hypotheses is that men and women differ in the way they divide attention across a visual stimulus regardless of its content. These studies showed the capacity of eye tracking to identify attractiveness and sexual desire in a non-paraphilic context. Given the above point, researchers have applied eye tracking in paraphilic contexts, especially with paedophilic interest. This literature review thus aims to summarize the use of eye tracking in the forensic context with child sexual offenders and show if eye tracking can discriminate subjects with paedophilic interests.

## Methods

In this systematic review, the PubMed, MEDLINE and Google Scholar databases were searched for quantitative studies published in English between 2012 and 2021 using the following key words: “eye movements”, “eye tracking” combined with “sexual offenders”, “sexual interest”, “paedophilic sexual interest”, “child sexual offenders”. Exclusion criteria were articles concerning eye tracking in a context other than of paedophilic interest, general studies about human sexuality and non-scientific studies (e.g. presentation of the establishment of assessment laboratories, overview of an evaluation of paedophilic sexual interest, case report). Reference lists of relevant publications were also hand-searched to retrieve additional sources. Abstracts were screened and the full texts of suitable studies were obtained. Study characteristics will be summarized narratively and in Table 1.

**Table 1.** Summary of the studies included in the literature review with samples, stimuli and results.

References	Sample (effectives)	Stimuli	Results
Fromberger et al. [36]	Three groups: paedophiles (*n =* 22); non-paedophilic forensic controls (*n =* 8); healthy controls (*n =* 52).	Pairs of pictures of children and adults at the same time (girl *vs.* woman, boy *vs.* man).	Paedophiles had a significantly longer viewing time for child stimuli (*P =* 0.037), a significantly shorter fixation latency for child stimuli (*P =* 0.002), and a significantly longer relative fixation time for child stimuli (*P =* 0.039). Receiver operating characteristic (ROC) analyses showed that fixation latency measurement discriminated between paedophiles and non-paedophiles with high accuracy (area under the curve, AUC = 0.902). Relative fixation time and viewing time also discriminated between paedophiles and non-paedophiles, (respectively AUC = 0.828 and AUC = 0.759)
Fromberger et al. [43]	Three groups: paedophiles (*n =* 22); forensic inpatients (*n =* 8); healthy controls (*n =* 52).	Pairs of pictures of children and adults at the same time (girl *vs.* woman, boy *vs.* man). Areas of interest were defined on each pictures (head, breasts, waist, and pubic region).	Paedophiles had a significantly shorter average entry time for the child pictures (*P =* 0.005); forensic and healthy controls had a significantly shorter average entry time for the adult pictures (respectively *P =* 0.021 and *P <* 0.001). Paedophiles showed the shortest entry time to the child’s head (*P <* 0.001), followed by the child’s pubic region and waist. Paedophiles showed a significantly longer relative fixation time for the child’s pubic region than other areas of interest (*P <* 0.001) but it is the same with adult’s pubic region (*P <* 0.001).
Jordan et al. [44]	Three groups: paedophiles (*n =* 22); forensic inpatients (*n =* 7); healthy controls (*n =* 50).	Pairs of three-dimensional cube figures and pictures of children (boys and girls) and adults (men and women). Rotation stimuli and one sexual distractor were showed in same time.	Paedophiles viewed sexual distractor (adult and child) significantly earlier than the forensic control group (*P =* 0.042) and paedophiles exhibited significant longer fixation latencies for mental rotation figures compared to both control groups (*P =* 0.009 with forensic control and *P <* 0.001 with non-forensic controls). Then, eye movements were computed in an attentional control index (ACI). Results indicate a significantly weaker control of eye movements in paedophiles compared to non-paedophiles. The ACI fixation latency with adult sexual distractor discriminated between paedophiles and non-paedophiles, sensitivity is hight (90.9%) and specificity is moderate (77.4%). With child sexual distractor, the ACI discriminated with hight accuracy between paedophiles and non-paedophiles, sensitivity and specificity are high (respectively 90.9% and 84.9%). ROC analysis with fixation time showed moderate accuracy with ACI for adult sexual distractor to discriminate paedophiles and non-paedophiles (sensitivity 71.9%, specificity 63.9%). With child sexual distractor, the ROC analysis discriminated with moderate accuracy between paedophiles and non-paedophiles (sensitivity 84.2% and specificity 63.6%).
Jordan et al. [45]	Three groups: paedophiles (*n =* 22); forensic inpatients (*n =* 7); healthy controls (*n =* 50).	Pairs of three-dimensional cube figures and pictures of children (boys and girls) and adults (men and women). Rotation stimuli and one sexual distractor were showed in same time.	Paedophiles exhibited shortest fixation latencies towards sexual distractor compared to both control groups, but it was only significant compared with the forensic control group (*P =* 0.042). Paedophiles differed from both control groups with longer relative fixation times to sexual distractor, but it was only significant compared to forensic controls (*P =* 0.014). Moreover, on age preference index (API) was computed, and ROC-analysis revealed that the API for the relative fixation time discriminated between paedophiles and non-paedophiles with moderate accuracy (AUC = 0.697, *P =* 0.007). The API differed between paedophiles and non-paedophiles with moderate sensitivity and specificity (respectively 71.9% and 63.6%).
Jordan et al. [46]	Three groups: outpatients with a sexual interest in children (*n =* 11); paedophilic forensic inpatients (*n =* 22); control group (*n =* 60) divided into eight forensic inpatients and 52 healthy subjects.	First step: pairs of pictures of children and adults at the same time (girl *vs.* woman, boy *vs.* man). Second step: sexual distractor task, one picture of character was showed with one pair of three-dimensional figures.	Outpatients with a sexual interest in children and paedophiles inpatients both viewed child stimuli earlier (only significant for paedophiles *P <* 0.05). Paedophiles inpatients viewed the child sexual distractor earlier (*P <* 0.05) and cognitive stimuli later than both other groups (*P <* 0.05).
Hall et al. [47]	Two groups: 13 child sexual offenders; 13 non offenders.	Pictures from Internet fashion catalogues, classified in pre-pubescent children, adults in their early 20s and adults in their late 30s or early 40s.	Child sexual offenders and non-offenders viewed adult stimulus without significant difference. With child pictures, child sexual offenders viewed more often toward the upper body of the female child figure than male child figure (*P =* 0.04). There is no significant difference in other regions, even with *post hoc* analyses.

## Results

Of 16 studies screened, only six articles met the inclusion criteria. Table 1 provides summary details of the included studies.

### Samples

The two studies by Fromberger et al. [36,43] compared three groups: 22 treated paedophiles; eight forensic patients (no paedophiles); and 52 healthy controls. Of note, the same sample population was used in both studies.

The first and the second studies of Jordan et al. [44,45] compared three groups: 22 paedophiles, seven forensic inpatients and 50 healthy subjects. The third study by Jordan et al. [46] compared three groups: 11 outpatients with a sexual interest in children; 22 inpatients with a sexual interest in children (same sample as Fromberger et al. [36,43]); and 60 individuals in the control group, which was divided into eight forensic inpatients and 52 healthy controls (same sample as Fromberger et al. [36,43]).

The study of Hall et al. [47] compared two groups: 13 child sexual offenders and 13 non-offenders’ subjects. Sexual offences ranged from downloading child pornography pictures to sexual intercourses.

### Stimuli and methodology

In the first study, Fromberger et al. [36] used pictures of children and adults and showed several paired images (girl and woman, boy and man) of each subject. Stimuli were selected from the Not-Real-People picture set featuring coloured pictures of nude and clothed male and female persons at five different stages of pubertal development (Tanner stages). Pictures of Tanner stages 1, 2, 4 and 5 were used; stages 1 and 2 defined a “child” category and 4 and 5 were combined into the ­category “adults”. Two dependent variables were selected: relative fixation time and fixation latency. Relative fixation time was defined as the sum fixation duration of all fixations located on the ­characters (boys, girls, woman, man) whereas fixation latency was defined as the duration from stimulus onset to the first fixation on character. After a test phase, each participant rated all stimuli with respect to sexual arousal and valence on a 9-point Likert scale (“unpleasant” to “pleasant”). In this study, the procedure is a free viewing task with unreal visual sexual stimuli.

In the second study, Fromberger et al. [43] used the same paired images but each figure on each stimulus was divided into four different areas of interests: head, breast, waist and pubic region. Relative fixation time and entry time were two dependent variables. Relative fixation time was defined as the sum fixation duration of all fixations located on the characters whereas the entry time was defined as the time from the stimulus onset to the first fixation on the area of interests. Following presentation of the paired images, each subject was asked: “Was one of these persons more attractive?”. Similar to the first study, each participant rated all stimuli with respect to sexual arousal and valence on the same 9-point Likert scale after the test phase. In this study, the procedure is a free viewing task with unreal visual sexual stimuli.

In the study of Jordan et al. [44], images from the Not-Real-People picture set were also used as sexual distractor stimuli. As in the studies by Fromberger et al. [36,43], pictures of Tanner stages 1 and 2 defined boys and girls categories and stages 4 and 5 defined men and women categories. In addition, pairs of three-dimensional cube figures were presented, identical and rotated or mirrored and rotated. Rotation stimuli and sexual distractor were showed in same time. Subjects had to say if the two figures were identical or not. Fixation latency and fixation time were two dependent variables. Fixation latency was defined as the duration from stimulus onset to the first fixation on three dimensional figures or character, whereas fixation time was the sum of fixation duration of all fixations on three dimensional figures or character. Eye movements were computed in an attentional control index (ACI) [44]. In this study, the procedure is a task irrelevant indirect measures of sexual interest with unreal visual sexual stimuli.

In the second study of Jordan et al. [45] stimuli are the same that in the previous study (pairs of three-dimensional cube figures and sexual distractor stimuli from Not-Real-People picture set). Subjects had to say if the two figures were identical or not. Rotation stimuli and sexual distractor were showed in the same time. Fixation latency and fixation time were two dependent variables defined like in the previous study [45]. In this study, the procedure is a task-irrelevant indirect measures of sexual interest with unreal visual sexual stimuli.

In the third study by Jordan et al. [46], images from the Not-Real-People picture set were also used. As in the studies by Fromberger et al. [36,43], pictures of Tanner stages 1 and 2 defined boy and girl categories and stages 4 and 5 defined men and women categories. In addition, cognitive stimuli were used in second tasks and presented as three-dimensional mental rotation figures (identical and rotated or mirrored and rotated). The experimental procedure was in two parts. The first step was the initial orientation to assess sexual interest. Several pairs of pictures appeared and after the presentation, the following question was asked: “Was one of these individuals sexually more attractive?”. The second step comprised the sexual distractor task. During this task, one picture of one of the four categories was shown with two three-dimensional figures. Subjects had to say if the two figures were identical or not [46]. Each three-dimensional figure was defined as an area of interest, as was each image of the different persons. The dependent variable was fixation latency (duration from stimulus onset to the first fixation). In this study, the procedure is a task-irrelevant indirect measures of sexual interest, with unreal visual sexual stimuli.

In the study of Hall et al. [47], images from Internet fashion catalogues were used. Figures were plain clothed and with neutral or low intensity happy facial expressions. Pictures were classified in three age groups: pre-pubescent children around 10, adults in their early 20s and adults in their late 30s or early 40s. There is no explanation about how facial expressions were evaluated and how character ages were determined. Furthermore, each body figure was divided into two different regions: the upper body, from the base of the neck to the end of the rib cage, and the waist-hip region, including stomach, hips and pubic region. But, in the results, authors described four regions (face, upper body, waist-hip and limbs). The number of fixation and viewing time were two dependant variables. The number of fixations and viewing time were normalised to the total number of fixations and total viewing time. Here, the procedure is a free viewing task with real static stimuli.

All protocols of these studies used static pictures of male and female at different stages of puberty. In the first five studies, images were used from the same picture set. All the dependant variables are not the same, the five first studies all used fixation latency, two of them used longer fixation time. The last study used number of fixation and viewing time. One on the most important difference about samples used, is that some studies used paedophiles subjects whereas, in Hall et al. [47] study, they used child sexual offenders, and we do not know if they suffer of paraphilic disorder. Protocols of the studies are summarized in Table 1.

### Findings

In the first study, Fromberger et al. [36] showed that paedophiles showed significantly shorter fixation latencies (*P =* 0.002) [36]. Receiver operating characteristic (ROC) analyses showed that fixation latency measurement discriminated between paedophiles and non-paedophiles with high accuracy (area under the curve [AUC] = 0.902). In addition, they demonstrated a significantly longer viewing time for child stimuli than forensic controls only (*P =* 0.037) and a longer relative fixation time for child stimuli than forensic and non-forensic controls (*P =* 0.039). Paedophiles had also a significantly shorter relative fixation time for adult stimuli than non-forensic and forensic controls (respectively *P* = 0.002 and *P =* 0.024). Relative fixation time and viewing time also discriminated between paedophiles and non-paedophiles, but with an AUC less important (respectively 0.828, high, and 0.759, moderate).

In the second study by Fromberger et al. [43], paedophiles demonstrated a significantly shorter average entry time for child pictures than for adult images (*P =* 0.005) [43]. By contrast, the average entry time was shorter with adult pictures for forensic and non-forensic controls (respectively *P =* 0.021 and *P <* 0.001). Pairwise comparisons concerning child stimuli revealed that paedophiles showed the shortest entry time to the child’s head (*P <* 0.001), followed by the pubic region and waist, which was not the case with forensic and non-forensic controls. With adult stimuli, paedophiles had the shortest entry time to the head (*P <* 0.001) and then the pubic region, whereas adult breasts were the shortest entry time after the head for both control groups. Concerning the relative fixation time, pairwise comparisons within child stimuli showed the child’s head as the region with the significantly longest time for all groups. Paedophiles spent a significantly longer relative fixation time looking at the child’s pubic region than breast and waist regions (*P <* 0.001). But they spend a significantly longer relative fixation time looking at the adult’s pubic region than breast and waist regions (*P <* 0.001). In both control groups, adult’s breast revealed significantly longer relative fixation time than adult’s waist or adult’s pubic region (*P <* 0.001 for each group).

In the first study of Jordan et al. [44], paedophiles viewed sexual distractor (adult and child) significantly earlier than the forensic control group (*P =* 0.042). Paedophiles exhibited significant longer fixation latencies for mental rotation figures compared to both control groups (*P =* 0.009 with forensic control and *P <* 0.001 with non-forensic controls).

Results indicate a significantly weaker control of eye movements in paedophiles compared to non-paedophiles. The ACI fixation latency with an adult sexual distractor discriminated between paedophiles and non-paedophiles, sensitivity is high (90.9%) and specificity is moderate (77.4%). With child sexual distractor, the ACI discriminated with high accuracy between paedophiles and non-paedophiles, sensitivity and specificity are high (respectively 90.9% and 84.9%). ROC analysis with fixation time showed moderate accuracy for adult sexual distractor to discriminate paedophiles and non-paedophiles (sensitivity 71.9%, specificity 63.9%). With child sexual distractor, the ROC analysis discriminated with moderate accuracy between paedophiles and non-paedophiles (sensitivity 84.2% and specificity 63.6%).

In the second study of Jordan et al. [45], paedophiles exhibited shortest fixation latencies towards sexual distractor compared to both control groups, but it was only significant compared with the forensic control group (*P =* 0.042). Paedophiles differed from both control groups with longer relative fixation times to sexual distractor, but it was only significant compared to forensic controls (*P =* 0.014). Moreover, an age preference index was computed (API), and ROC-analysis revealed that the API for the relative fixation time discriminated between paedophiles and non-paedophiles with moderate accuracy (AUC = 0.697, *P =* 0.007). The API differed between paedophiles and non-paedophiles with moderate sensitivity and specificity (respectively, 71.9% and 63.6%).

In the third study by Jordan et al. [46], outpatients and inpatients both viewed child stimuli earlier and adult stimuli later than the control group, but it was only significant between inpatients and controls (*P <* 0.05). Inpatients viewed the child sexual distractor earlier than both other groups (*P <* 0.05) and cognitive stimuli later than other groups (*P <* 0.05).

In the study of Hall et al. [47], child sexual offenders and non-offenders viewed adult stimulus without significant differences. With child pictures, child sexual offenders viewed more often toward the upper body of the female child figure than male child figure (*P =* 0.04). There is no significant difference in other regions, even with *post hoc* analyses.

Significant results of the five studies included in a large project, with same samples, and lead by the same team are identical in terms of fixation latency, and for the two studies of Fromberger et al. [44, 45] in terms of longer fixation time. At the studies progress, we observed the creation of different indexes as ACI or API. These indexes showed high and moderate characteristics and provide evidence for the usefulness of eye tracking. For the moment, cut offs of these indexes does not seem to be ­clinically relevant. The sixth study analysed number of fixations and there are no significant differences between the two groups on child pictures [47]. The most relevant results are summarized in Table 1.

## Discussion

This review suggests that fixation latency and longer fixation are interesting measures with eye tracking and can discriminate between paedophilic and non-paedophilic interest. Compared to the control groups, paedophiles seemed to have a shorter fixation latency and a longer fixation time for child stimuli. With adult stimuli, results are less significant. When pictures are divided in different areas of interest, only one study showed a significant longer relative fixation time for pubic region on child pictures for paedophile subjects; this difference is not observed with number of fixations, and it is not specific because the same results is observed with adult pictures.

There are several limitations inherent to the results of the included studies in that the number of cases is relatively low and samples are not so various. Moreover, most of the paedophiles included were treated and it is probably that their acceptance of their paraphilic disorder was better than that of subjects who deny their own paedophilia. Moreover, many studies used the same images to stimulate subjects. Thus, it could be interesting to compare different stimuli and identify most discriminants. In fact, several studies, not in paraphilic context, showed that the background of visual stimuli can influence attentional processing. The degree to which nonsexual contextual cues attract attention seems unclear [48,49]. Most of the time, static images are used with eye tracking. In non-paraphilic context, Tsujimura et al. [42] used dynamic video stimuli and observed interesting and new gender difference in specificity of visual attention. Finally, some studies used task-irrelevant indirect measures whereas researchers showed that task-relevant measures should be superior because in this type of task, stimuli cannot be underrate [50,51]. We should use a same group of paedophiles subjects and submit them to different stimuli. Only one aspect of stimuli could be modified each time to accurately assess the influence of this change on eye tracking results. In this project of study design, paedophiles subjects would be their own control.

Pictures chosen were non-erotic and non-pornographic and it is possible that they were insufficiently stimulating, which could lead to an increase in false negatives. Even in research context, the use of more explicit pictures could be limited and forbidden by each country laws and assimilated to child pornography.

Overall, these results are interesting and support some research hypotheses despite contradictory results [36,43,46,47]. We can perceive the potential of eye tracking in the diagnostic procedure related to sexological assessment in various contexts. But contrary to what some studies suggests, and especially Fromberger et al. [36], actually eye tracking cannot diagnose paedophilia, because eye tracking can only consider, at least in part, the presence or not of criterion A according to DSM-5 [10]. Nevertheless, irrespective of the context of use, the diagnosis of paedophilia is very stigmatizing and can have several consequences on the life of the subject (family, social, professional, etc.). This impact is all the more important in a forensic context, especially when a diagnosis of paedophilia is made during a pre-trial psychiatric assessment where there is presumption of innocence. Thus, the question can be asked as to the degree of importance that should be given to additional tests in the diagnostic procedure. In forensic context with accusations of child pornography without sexual contact (or suspicion of contact) with children, eye tracking seems to be unhelpful for diagnosis, because these charges may be sufficient to meet diagnostic criterion A. Eye tracking would be reserved for cases of accusations of sexual acts with children (with contacts).

Moreover, eye tracking may be relevant in other contexts—treatment, risk assessment, risk management [52]. The influence of treatments and sexual therapies on paedophilic interests could be highlighted and measured. For this, we should compare a group of paedophiles subjects cared and a group of paedophile subjects non-cared. It could be a sign more objective of improvement that could influence dangerousness and allow a change of penali care. Neurosciences are increasingly used in psychiatry and forensic psychiatry. In addition, results of neuroimaging examinations are sometimes at the heart of Court debates, which also raise ethical and societal issues [53–55].

Further studies with other subject samples and design methodologies should be performed in order to examine the validity of eye tracking to discriminate between paedophiles and non-paedophiles among child sexual offenders. Notably, the use of this technique should respect some ethical concerns to avoid drifts.

## Conclusion

Eye tracking is a very interesting tool to evaluate sexual attractiveness by attentional processes. However, despite a certain enthusiasm for the technique in the context of the evaluation of sexual offenders, there are very few specific studies of interest regarding its use to discriminate paedophiles among child sexual offenders. Results of the studies included in this review suggest interesting ways to identify paedophiles among child sexual offenders, but further research with larger and different sample groups, by different research teams, are necessary to confirm these findings. One of the difficulties of this type of research area is to include volunteer paedophiles and use adapted stimuli.

## Authors’ contributions

Tony Godet and Gérard Niveau wrote the draft and revised the manuscript. Both authors contributed to the final text and approved it.
